# Selection Pressure Pathways and Mechanisms of Resistance to the Demethylation Inhibitor-Difenoconazole in *Penicillium expansum*

**DOI:** 10.3389/fmicb.2018.02472

**Published:** 2018-10-31

**Authors:** Emran Md Ali, Achour Amiri

**Affiliations:** Department of Plant Pathology, Tree Fruit Research and Extension Center, Washington State University, Wenatchee, WA, United States

**Keywords:** demethylation inhibitors, blue mold, *CYP51*, postharvest, fludioxonil, overexpression

## Abstract

*Penicillium expansum* causes blue mold, the most economically important postharvest disease of pome fruit worldwide. Beside sanitation practices, the disease is managed through fungicide applications at harvest. Difenoconazole (DIF) is a new demethylation inhibitor (DMI) fungicide registered recently to manage postharvest diseases of pome fruit. Herein, we evaluated the sensitivity of 130 *P. expansum* baseline isolates never exposed to DIF and determined the effective concentration (EC_50_) necessary to inhibit 50% germination, germ tube length, and mycelial growth. The respective mean EC_50_ values of 0.32, 0.26, and 0.18 μg/ml indicate a high sensitivity of *P. expansum* baseline isolates to DIF. We also found full and extended control efficacy *in vivo* after 6 months of storage at 1°C. We conducted a risk assessment for DIF-resistance development using ultraviolet excitation combined with or without DIF-selection pressure to generate and characterize lab mutants. Fifteen DIF-resistant mutants were selected and showed EC_50_ values of 0.92 to 1.4 μg/ml and 1.7 to 3.8 μg/ml without and with a DIF selection pressure, respectively. Resistance to DIF was stable *in vitro* over a 10-week period without selection pressure. Alignment of the full *CYP51* gene sequences from the three wild-type and 15 mutant isolates revealed a tyrosine to phenylalanine mutation at codon 126 (Y126F) in all of the 15 mutants but not in the wild-type parental isolates. Resistance factors increased 5 to 15-fold in the mutants compared to the wild-type-isolates. DIF-resistant mutants also displayed enhanced *CYP51* expression by 2 to 14-fold and was positively correlated with the EC_50_ values (*R*^2^ = 0.8264). Cross resistance between DIF and fludioxonil, the mixing-partner in the commercial product, was not observed. Our findings suggest *P. expansum* resistance to DIF is likely to emerge in commercial packinghouse when used frequently. Future studies will determine whether resistance to DIF is qualitative or quantitative which will be determinant in the speed at which resistance will develop and spread in commercial packinghouses and to develop appropriate strategies to extend the lifespan of this new fungicide.

## Introduction

The extended storage of apple fruit for up to 12 months in low temperatures and controlled atmospheres (low O_2_ and high CO_2_ concentrations) makes them prone to infections by several fungal pathogens. *Penicillium expansum* is an ascomycete fungus causing blue mold, a major postharvest disease of apple and pear fruit worldwide (Amiri and Bompeix, 2005a; Morales et al., 2007; Jurick et al., 2011). In recent surveys in Washington State, blue mold accounted for nearly 50% of total decay caused on apple postharvest (Amiri and Ali, 2016). *Penicillium expansum* is a typical airborne and wound pathogen with short life cycles and copious asexual conidial production which are responsible for pome fruit infections in storage rooms (Sanderson and Spotts, 1995; Amiri and Bompeix, 2005a). Spores of *P. expansum* seldom infect fruit in orchards (Amiri and Bompeix, 2005a) but can be abundant on storage bins and in storage rooms if appropriate sanitation practices are not implemented at the beginning of the season (Spotts and Cervantes, 1993; Sanderson and Spotts, 1995; Amiri and Bompeix, 2005a). Primary infections, resulting from residual inoculum, may start on fresh wounds or punctures caused at harvest or during postharvest handling (Rosenberger et al., 1991; Amiri and Bompeix, 2005b). Thereafter, inoculum can quickly build up inside storage rooms to cause multiple secondary infections (Amiri and Bompeix, 2005a).

There is no known host resistance to *P. expansum* in current commercial apple cultivars. Therefore, besides some sanitation practices at packing facilities and other biological or physical methods with moderate efficacy, management of *P. expansum* and other postharvest pathogens is mainly achieved using single-site synthetic fungicides. The number of molecules registered postharvest has been limited to three, i.e., thiabendazole (TBZ) registered four decades ago, pyrimethanil (PYR) and fludioxonil (FDL) registered 15 years ago. *P. expansum* is considered a “high risk” fungus for fungicide resistance development. Thus, resistance to TBZ, linked to several mutations in the β-tubulin gene, has been reported widely from numerous production regions worldwide (Rosenberger et al., 1991; Errampalli et al., 2006; Malandrakis et al., 2013; Yin and Xiao, 2013). Resistance to PYR has emerged in recent years in the U.S. Pacific Northwest and Mid-Atlantic regions but remains at relatively low frequencies (Jurick et al., 2017; Caiazzo et al., 2014; Yan et al., 2014; Amiri et al., 2018). Lately, low levels of resistance or reduced sensitivity to FDL have been sporadically found in some U.S. apple packinghouses (Gaskins et al., 2015; Amiri et al., 2017). The emergence of resistance to PYR and FDL and the relatively lower FDL efficacy against *Neofabraea* spp. (Amiri, unpublished data) suggest registration of new fungicides with different modes of action than the current three postharvest fungicides is necessary to maintain effective disease control.

Difenoconazole (1-[2-[2-chloro-4-(4-chloro-phenoxy)-phenyl]-4-methyl[1,3]-dioxolan-2-ylmethyl]-1H-1,2,4-triazole) (Supplementary Figure S1), a new demethylation inhibitor (DMI) fungicide, was registered in 2016 for postharvest use in pome fruit. It is pre-mixed with FDL and commercially available as Academy™ (Syngenta Crop Protection). Difenoconazole (DIF) has a systemic activity and broad-spectrum antifungal potency as shown recently (Hof, 2001; Fonseka and Gudmestad, 2016; Bartholomäus et al., 2017; Dang et al., 2017; Jurick et al., 2017; Koehler and Shew, 2018; Ali et al., 2018). DMIs, such as DIF, target the sterol 14α-Demethylase Cytochrome P450 (*CYP51*), an essential component of fungal membrane sterols required for a proper membrane functioning (Rodriguez et al., 1985; Joseph-Horne and Hollomon, 1997). Although classified as “medium risk,” resistance to DMIs has been reported in several fungal pathogens (Golembiewski et al., 1995; Erickson and Wilcox, 1997; Schnabel and Jones, 2001; Fraaije et al., 2007; Omrane et al., 2015), i.e., in *Penicillium digitatum* from citrus fruit (Eckert and Ogawa, 1988; Bus et al., 1991; Hamamoto et al., 2001a; Ghosoph et al., 2007; Sun et al., 2011). Resistance to the DMIs in *P. digitatum* and other micro-organisms has been linked to single amino-acid alterations in the target site (Délye et al., 1997; Favre et al., 1999; Diaz-Guerra et al., 2003; Leroux et al., 2007; Wang et al., 2015; Pereira et al., 2017), increased energy dependent fungicide efflux mechanisms (Nakaune et al., 1998; Reimann and Deising, 2005), or overexpression of the *CYP51* gene (Van Den Brink et al., 1996; Hamamoto et al., 2001a; Schnabel and Jones, 2001; Sun et al., 2013). A mechanism involving both amino-acid alterations with overexpression of the *CYP51* gene has been suggested to cause DMI resistance in some other fungi (Mellado et al., 2007; Snelders et al., 2008; Mair et al., 2016; Lichtemberg et al., 2017).

The widespread resistance of *P. expansum* to two of the three existing postharvest fungicides and the fact that DIF will be premixed with FDL for which some tolerance has already been reported suggest a thorough risk assessment is needed before DIF becomes widely used. Herein, we evaluated the sensitivity of a baseline wild-type *P. expansum* population to DIF, determined impact of storage conditions on fungicide potency, and evaluated the risk and mechanisms of resistance development to DIF in lab mutants of *P. expansum*. We show that DIF would be a useful tool to include in future management programs but strategies are needed to extend its lifespan.

## Materials and Methods

### Cultivation and Characterization of *P. expansum* Baseline Isolates

A total of 130 *P. expansum* isolates, never exposed to difenoconazole (DIF), collected in 2004 and 2005 were used to determine the baseline sensitivity. These isolates were single-spored and stored in 20% glycerol at -80°C at the WSU-TFREC pathology laboratory. Isolates were identified to the species level based on the β-*tubulin* gene using the PE-Chang5′-F and PE-Tub-R2 primer pair (Supplementary Table S1) developed in this study and based on a previous work published by Sholberg et al. (2005). Prior to each experiment, the isolates were grown on potato dextrose agar (PDA) at 22°C for 5 to 7 days or until profuse sporulation was observed. Three wild-type isolates, Pe3175, Pe3136, and Pe3334, were used for mutant selection as described below.

### Fungicides

Formulated difenoconazole (DIF, Thesis, Syngenta Crop Protection, Greensboro, NC, United States), fludioxonil (Scholar SC, Syngenta), and fluioxonil + difenoconazole (Academy, Syngenta) were used in this study. For *in vitro* bioassay, stock solutions of 1,000 μg/ml of the active ingredients were made in sterile distilled water and stored in the dark at 4°C for no more than 21 days. Preliminary tests showed no negative effect of sensitivity levels of fungicide stocks prepared and stored as described above (data not shown). DIF was used to determine the baseline sensitivity of the 130 baseline isolates and selected mutants, whereas fludioxonil (FDL) was used to determine cross-sensitivity with DIF in mutant isolates. For *in vivo* assays, the formulated products were used following the label rate or as otherwise described.

### Determination of Baseline Sensitivity to Difenoconazole

The sensitivity of 130 baseline isolates to DIF was determined *in vitro* using mycelial growth, spore germination, and germ tube inhibition assays on 1% malt extract agar (MEA) medium. Molten autoclaved MEA was cooled to 50°C and DIF was added from the stock to obtain final concentrations of 0.0, 0.05, 0.1, 0.5, 5.0, and 10.0 μg/ml and poured into 60-mm Petri plates. Spores were harvested from 7-day-old plates by transferring dry spores with a sterile plastic loop to a 2-ml tube containing 1 ml of sterile deionized water with 0.05% Tween 20. The spore concentrations were determined with a hemacytometer and adjusted to 10^5^ spores/ml. A 10 μl-droplet was plated onto the center of a DFC-amended and non-amended MEA plates, which were incubated for 6 days at 20°C before measuring the colony diameter. The spore germination inhibition assay was conducted on MEA as described for mycelial growth except that spore germination was measured microscopically after 16 h incubation at 20°C. A conidium was considered germinated when the germ tube length was at least twice the conidium diameter. The germ tube length of 10 conidia per plate was measured using the reticle ruler and used to assess sensitivity based on germ tube length inhibition. For each bioassay, trials were conducted in quadruplicates and repeated twice.

### Generation and Characterization of DIF-Resistant Mutants

#### Mutants Generation

Three *P. expansum* wild-type isolates, Pe3175, Pe3136, and Pe3334, were used as parental isolates to generate fungicide-resistant mutants using ultraviolet (UV) light excitation as described by Li and Xiao (2008) with some modifications. For each isolate, a 100 μl-aliquot of a spore suspension at 3.7 × 10^7^ conidia per ml was spread onto PDA media amended with 10 μg/ml DIF. Five replicate-plates were used for each of the three isolates and the plates were incubated at 20°C for 5 h in the dark before exposition to UV light (plates 27 cm from the UV light at 253.7 nm) for 30 s followed by a 7-day- incubation in the dark at 20°C. Growing colonies, including from the non-UV exposed WT parental isolates, were transferred twice on PDA amended with DIF at 10 μg/ml and incubated for 6 days for each transfer. Thereafter, colonies were grown and transferred twice on DIF-free PDA and incubated at 20°C. No growth was observed on the WT isolates grown on PDA with 10 μg/ml after 12 days of incubation (data not shown).

#### Evaluation of Resistance of Mutants to DIF and Its Stability With and Without Selection Pressure

Five colonies were selected from each wild-type parent and the total of 15 mutants were characterized for their sensitivity to DIF using a mycelial growth assay as described above for the baseline isolates. The stability of resistance to DIF was evaluated without and with DIF selection pressure. Nine isolates, i.e., three WT-isolates Pe3136, Pe3175, and Pe3334 and three mutants selected from each of them, were used. A 10-μl droplet of a spore suspension (10^4^ spores/ml) of each isolate was transferred to fresh free-DIF MEA plates (three replicates/isolates) to test for stability in absence of selection pressure or onto MEA plates amended with DIF at 2.5 μg/ml to test with selection pressure. Isolates were incubated for 1 week at 20°C, then transferred weekly on DIF-free or DIF-amended plates for seven additional successive weeks. After 8 weeks, the EC_50_ values were determined based on a mycelial growth inhibition assay and compared with the initial EC_50_ values of the WT and the mutant isolates.

#### Virulence on Apple Fruit and Efficacy of DIF to Control Resistant Mutants

Organic cv. Fuji apples harvested at commercial maturity from an experimental orchard in East Wenatchee, Washington, were surface-disinfected for 3 min in 0.8% sodium hypochlorite, rinsed twice with sterile water and air-dried. Fruits were punctured twice near the stem-end area with a sterile needle (1.5 mm diameter, 3 mm deep), dipped for 30 s in a suspension of formulated DIF (Thesis, Syngenta) at label rate of 0.26 mg/L, allowed to dry at 4°C for 12, 24, 48, and 96 h then inoculated with a 25-μl droplet of spore suspension (5 × 10^4^ spores/ml) on each wound. Control fruit were wounded and dipped in sterile water. The three WT-baseline isolates, Pe3334 (EC_50_ = 0.13 μg/ml), Pe3136 (EC_50_ = 0.21 μg/ml), and Pe3175 (EC_50_ = 0.29 μg/ml), and nine lab DIF-mutants that had EC_50_ values ranging from 0.6 to 3.7 μg/ml were used for inoculation. Eight replicate fruit in duplicate were used for each isolate and fungicide combination and the trials were conducted twice. Inoculated fruit (two reps of four fruit each per each treatment) were incubated in separate sterile boxes in saturated growth chambers at 0°C and a regular atmosphere. Disease incidence and severity were determined relative to untreated control fruit monthly up to 6 months of storage.

#### Cross-Sensitivity With Fludioxonil and Efficacy of Academy™ to Control DIF-Resistant Mutants

Difenoconazole is pre-mixed with fludioxonil (FDL) and registered as Academy™ for commercial use. Therefore, we verified that the DIF-lab mutants were not resistant to FDL. The three parental wild-type isolates, Pe3136, Pe3175, Pe3334, and the 15 DIF-resistant mutants selected were tested on PDA amended with FDL at 0, 0.001, 0.01, 0.1, 1.0, and 10.0 μg/ml. Sensitivity tests were conducted based on a mycelial growth assay as described above for the baseline isolates. Three replicate plates were used for each isolate and fungicide concentration and the experiment was repeated twice.

A detached fruit assay was conducted to evaluate the efficacy of the mixture DIF + FDL (Academy™, Syngenta) to control DIF-resistant mutants. Organic Fuji apples were prepared as described above for virulence assay and treated preventively with Academy at 1.25 ml/L (0.26 mg DIF/L), then inoculated 4 h later with spore suspensions at 5 × 10^4^ spores/ml of the parental wild-type isolate Pe3175 and two of its mutants. The number of replicate fruit, storage conditions and assessments of disease incidence and severity were conducted similarly to virulence assays above.

### Amplification and Sequencing of the *PeCYP51* Gene

DNA of the three WT parental isolates and 15 mutants, i.e., 5 mutants from each WT isolate, was extracted from DIF-free 14 day-old-PDA plates using the FastDNA Kit (MP Biomedicals, Solon, OH, United States) according to the supplier’s instructions. The quantity and purity of DNA was measured with a NanoDrop Spectrophotometer (ND-1000, NanoDrop Technology, Wilmington, DE, United States). Three sets of primers were developed (Supplementary Table S1) based on the sequences of *CYP51* in GenBank accession numbers XM016737741 and NW015971309 (Supplementary Figure S2) and used to amplify 1751 bp of the *P. expansum CYP51* (*PeCYP51*) gene. The primer pair CYP51-S2F/CYP51-S2R, developed based on the GenBank accession XM016737741, was used to amplify a 567 bp fragment of the coding region. The two other sets of primers were developed to amplify parts of the *CYP51* and the flanking regions based on the GenBank accession NW015971309. The primer set CYP51-S5′F/CYP51-S1R was used to amplify an 811 bp fragment including 137 bp upstream of the 5′ end of *CYP51* whereas the set CYP51-S3F/CYP51-S3′R was used to amplify an 884 bp fragment including 129 bp downstream the 3′ end of the *CYP51* gene through conventional PCR. Fungal DNA (100 ng) was used as a template for PCR reactions which were run in 30 cycles of 94°C (30 s), 55°C (60 s), and 72°C (60 s) in a Bio-Rad T1000 thermocycler using EconoTaq^®^ plus green 2× master mix (Lucigen, Middleton, WI, United States) following a protocol suggested by supplier. All PCR products were analyzed by electrophoresis on a 1% agarose gel, purified using a PCR purification kit (Qiagen, Valencia, CA, United States), and Sanger-sequenced at Retrogen, Inc. (San Diego, CA, United States). Sequences from the three fragments of the gene were concatenated and a multiple alignment was constructed using BioEdit Version 7.2.5 (Hall, 1999) to determine nucleotide and amino acid changes.

### RNA Extraction and Quantitative Expression of the *PeCYP51* Gene

Total RNA was isolated from DIF-free 14-day-old PDA plates using a ZR Fungal/Bacterial RNA MiniPrep Kit (Zymo Research, Irvine, CA, United States) according to the supplier’s instructions. All RNA was analyzed for quantity and quality spectroscopically on 1% TBE agarose gel. After extraction, 1 μg of total RNA from each sample was treated with DNase and single strand cDNA was synthesized using the Bio-Rad iScript™ gDNA Clear cDNA Synthesis Kit (Bio-Rad Inc., Hercules, CA, United States). All samples were DNase-treated and cDNA synthesized in a single run with one batch of reagents and stored at -80°C. All quantitative (qPCR) reactions were run on a CFX96™ Real-Time PCR Detection System using SsoAdvanced™ Universal SYBR^®^ Green Supermix (Bio-Rad Inc., Hercules, CA, United States) in a 10 μl-reaction volume containing 5 μl of SYBR Green Supermix (antibody-mediated hot-start Sso7d fusion polymerase, 50 mM Na^+^, 1.5 mM Mg2^+^, 1.2 mM dNTPs, and 250 nM annealing oligo), 0.3 μl of 1000 nM of each forward (cyp51A-F/β-actin-F) and reverse primers (cyp51A-R/β-actin-R) (Table 1), and 2 μl (10 pg) of cDNA and 2.4 μl of PCR grade water. The recommended thermal cycling protocol for SsoAdvanced™ SYBR Green was used at an annealing/extension temperature of 60°C, and a melt curve analysis was included. The CFX Maestro™ Software ^1^ was used to analyze all qPCR data. The 2^-ΔΔCt^ equation (Livak and Schmittgen, 2001) was used to calculate the relative gene expression using the ß-actin as a reference control gene. *CYP51* expression data presented herein are averages of nine values for each isolate across three separate experimental runs. The “sample maximization” experimental set-up for multi-plate qPCR studies was used to minimize technical variation between samples (Hellemans et al., 2007).

**Table 1:** Virulence of wild-type and mutants of *Penicillium expansum* on detached Fuji apples and *in vivo* control efficacy of preventive difenoconazole applications.

	Blue mold incidence (%) and lesion diameter (mm) on fruit treated or not with DFC								
	UV-C treatment^a^				8-Weeks selection pressure^b^			
			Lesion diameter (mm)				Lesion diameter (mm)	
Isolate	Mean EC_50_	Incidence^d^	DFC^-C^	DFC^+^	Mean EC_50_	Incidence	DFC^-^	DFC^+^
Pe3334-WT (0.13)^e^	…	0.0^∗^	55.8	0.0^∗^	…	0.0^∗^	39.5	0.0^∗^
Pe3334-Ml	1.3	75.0	54.5	18.5	1.7	100	36.8	14.5
Pe3334-M2	1.4	75.0	49.5	11.5	1.8	100	38.3	14.8
Pe3334-M3	1.4	100.0	52.5	7.5^∗^	1.9	100	38.8	15.3
Pe3136-WT (0.21)	…	0.0^∗^	53.8	0.0^∗^	…	0.0^∗^	40.3	0.0^∗^
Pe3136-Ml	1.3	75.0	53.8	22.5	1.9	100	37.3	16.5
Pe3136-M2	1.2	75.0	52.3	15.8	1.7	100	38.5	17.0
Pe3136-M3	1.3	75.0	51.8	18.3	1.9	100	39.8	17.8
Pe3175-WT (0.29)	…	0.0^∗^	55.8	0.0^∗^	…	0.0^∗^	40.8	0.0^∗^
Pe3175-Ml	2.5	100.0	52.5	34.3	3.7	100	39.3	18.3
Pe3175-M2	2.5	75.0	49.5	21.8	3.7	100	39.8	17.3
Pe3175-M3	2.4	75.0	54.5	23.8	3.6	100	38.8	16.8

*^a,b^Indicate mutants selected after UV excitation and UV + 8 weeks of selection pressure on difenoconazole (DIF)-amended MEA, respectively. ^C^- and +indicate virulence (lesion diameter in mm) on untreated (control) and DIF-treated fruit, respectively. ^d^Blue mold incidence expressed as the number of infected fruit relative to the total number of fruit inoculated after 6 months of storage at 1°C. ^e^Numbers in brackets indicate effective concentration to inhibit 50% growth (EC_50_ in μg/ml) of the wild-type (WT) isolates. Values within the same column followed by an asterisk are statistically different from the other values based on an ANOVA test and Student’ t-test at P ≤ 0.05.*

### Statistical Analysis

Data from the two independent runs of *in vitro* and *in vivo* experiments were averaged when no statistical difference was observed between the two runs. Difenoconazole *in vitro* sensitivity data, expressed as percent inhibition relative to the control, were computed and log-transformed to calculate effective concentrations to inhibit 50% growth or germination (EC_50_). Variation factors (VF) were calculated as the highest EC_50_ value by the lowest EC_50_ value within the baseline population, whereas the resistance factors (RF) for DIF-mutants were calculated as their EC_50_ value by the EC_50_ value of the parental WT-isolate. Virulence *in vivo* bioassay data were used to calculate disease incidence and severity. Gene expression was expressed as the ratio between *CYP51* and β-actin genes. Data were subjected to ANOVA analyses and mean separations using Student *t*-test at *P* < 0.05 in SAS software (Version 9.2, SAS Institute Inc., Cary, NC, United States).

## Results

### *In vitro* and *in vivo* Sensitivities of *P. expansum* Baseline Isolates to Difenoconazole

**FIGURE 1:**
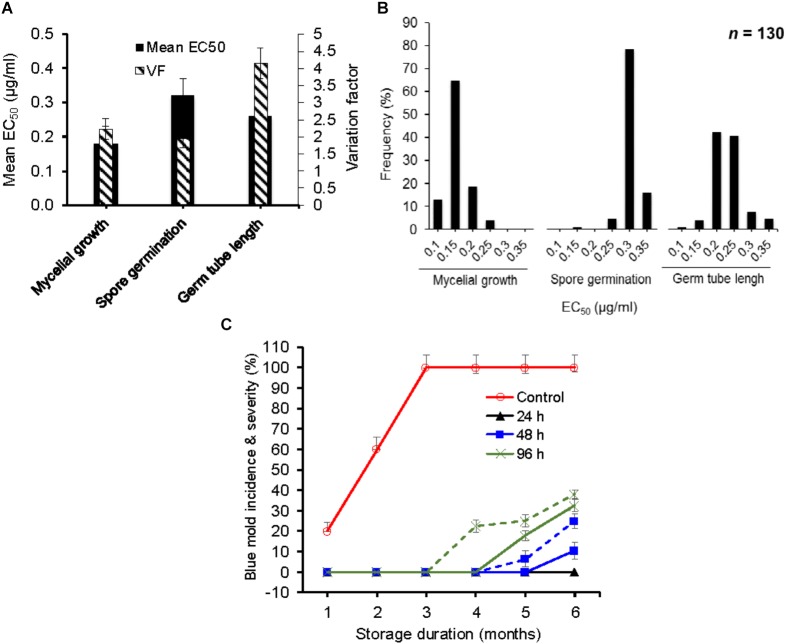
*In vitro* and *in vivo* efficacy of difenoconazole against baseline isolates of *Penicillium expansum.* **(A)** Mean effective concentration of difenoconazole for 50% growth, germination and germ tube elongation inhibition (EC_50_) and variation factor (VF, highest EC_50_/lowest EC_50_) of 130 baseline isolates of *P. expansum.* **(B)** Frequency distribution of respective EC_50_ values among 130 baseline isolates of *P. expansum.* **(C)** Temporal change in blue mold incidence (continuous lines) and severity (dashed lines) on detached apple fruit treated preventively with 0.26 mg/L of difenoconazole and inoculated with WT-isolates of *P. expansum* 24, 48, and 96 h post-treatment at 1°C and regular atmosphere. Data are the mean incidences from 3 WT-isolates (data were merged after no significance difference was observed between the isolates) and 58 fruit across two experimental runs. Data from the 12-h post-treatment inoculation are not shown because no disease was seen.

The mean EC_50_ values (±SD) for difenoconazole (DIF) determined for mycelial growth, spore germination and germ tube length inhibition were 0.17 ± 0.03, 0.32 ± 0.02, and 0.26 ± 0.06 μg/ml, respectively (Figure 1A). The EC_50_ values ranged from 0.13 to 0.29 μg/ml based on mycelial growth and from 0.19 to 0.37 μg/ml for spore germination inhibition and from 0.14 to 0.58 μg/ml for germ tube length inhibition (Figure 1B). The VF were 2.23, 1.95, and 4.14 μg/ml, respectively. The frequency distribution of EC_50_ values for germ tube length and mycelial growth were the closest to a unimodal distribution with a right-hand tail (Figure 1B), contrary to spore germination, which had a left-hand tail.

The EC_50_ value of the *P. expansum* WT-isolates did not affect the efficacy of DIF *in vivo* since the three isolates Pe3175 (EC_50_ = 0.13 μg/ml), Pe3334 (EC_50_ = 0.21 μg/ml), and Pe3175 (EC_50_ = 0.29 μg/ml) resulted in similar incidence and severity on detached fruit treated by the label rate of DIF. Therefore, data of the three isolates were averaged and presented in Figure 1C. The wild-type isolates were fully controlled on detached fruit for up to 6 months of storage at 0°C when DIF was applied 12 to 24 h pre-inoculation and for up to 4 months when DIF was applied preventively 48 or 96 h pre-inoculation (Figure 1C). After 6 months of storage, the blue mold incidence was 25 and 38% on fruit inoculated 48 and 96 h pre-inoculation, respectively, and similar severity trend was observed.

### Characterizations of DIF-Resistant Mutants

#### Resistance Levels, Stability, Cross-Resistance With FDL and Efficacy of DIF *in vivo*

**FIGURE 2:**
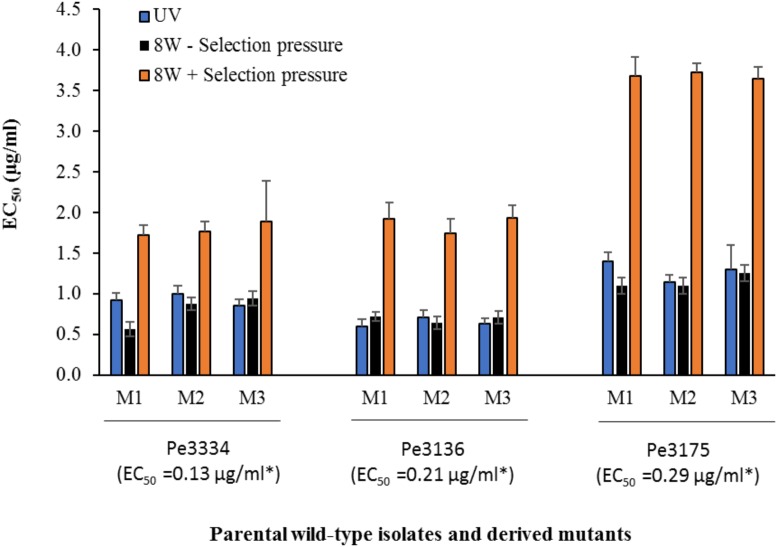
Mean effective concentration for 50% growth inhibition values (EC_50_) of difenoconazole for DIF-resistant mutants selected following UV excitation of Pe3334, Pe3136, and Pe3175 *P. expansum* isolates or after 8 weeks of adaptation on MEA amended with DIF at 2.5 μg/ml (+ selection pressure, orange bars) or without (– selection pressure, black bars) at 22°C. Asterisks indicates the EC_50_ values of each parental WT-isolate. Data are the mean of 12 values across two experimental runs for each mutant.

In total, 15 *P. expansum* mutants generated through UV excitation were tested for sensitivity to DIF using a mycelial growth inhibition assay to determine variation in their EC_50_ values compared to the parental wild-type (WT) isolates. The mutants selected from the parental WT isolates Pe3334 (EC_50_ = 0.13 μg/ml) and Pe3136 (EC_50_ = 0.21 μg/ml) had EC_50_ values ranging from 1.1 to 1.4 and respective RF ranging from 5.7 to 10.6, whereas the mutants selected from the parental isolate Pe3175 (EC_50_ = 0.29 μg/ml) had EC_50_ values ranging from 2.1 to 2.5 (Table 1) and RFs from 7.7 to 8.8. After 8 weekly transfers on MEA supplemented with DIF at 2.5 μg/ml (selection pressure), the EC_50_ values of the mutants ranged from 1.6 to 1.9 μg/ml for the mutants selected from the Pe3334 and Pe3136 WT isolates and from 3.3 to 3.7 μg/ml for the mutants selected from the parental isolate Pe3175 (Figure 2). RF values relative to the WT isolates ranged from 8 to 13 and increased by 1 to 1.5-fold relative to the first mutants transfer (data not shown). After 10 weekly transfers on DIF-free MEA (no selection pressure), the EC_50_ values of DIF-resistant mutants decreased by 0.04 to 0.36 μg/ml for 6 mutants out of nine tested while EC_50_ increased in three mutants. However, EC_50_ values were not significantly different from the first transfer and remained within the resistance range (Figure 2).

**FIGURE 3:**
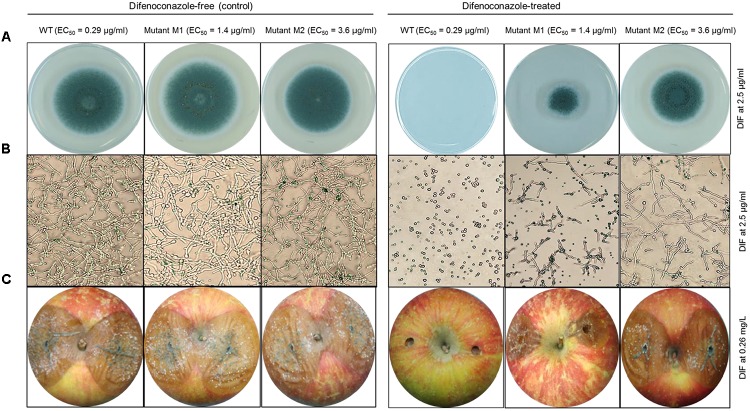
*In vitro* and *in vivo* efficacy of difenoconazole against *P. expansum* wild-type and DIF-mutants. **(A)** Mycelial growth and **(B)** germination of the *P. expansum* Pe3175 WT-isolate, mutant Ml (generated from UV-C treatment) and mutant M2 (UV-C + 8 weeks adaptation on DIF at 2.5 μg/ml) on DIF-free MEA (left, control) or on MEA amended with DIF at 2.5 μg/ml (right) after 24 h and 7 days incubation at 22°C for spore germination and mycelial growth, respectively. **(C)** Blue mold lesion diameter caused by Pe3175 WT and its respective mutants Ml and M2 on Fuji apples treated or not (control) with 0.26 mg/L DIF and incubated at 1°C for 6 months.

All the nine mutants originating from UV excitation caused blue mold on detached apple fruit and they were as virulent (lesion diameter) as the parental WT isolates (Table 1). While the three WT isolates were fully controlled by a preventive DIF application after 6 months of storage at 1°C (Table 1), the fungicide failed to control the nine selected mutants as the blue mold incidence ranged from 75 to 100% (Table 1 and Figure 3). The UV mutants adapted on 2.5 μg/ml of DIF for 8 weeks caused 100% blue mold incidence and their virulence (lesion diameter) was not significantly reduced compared to same isolate at the first transfer (Table 1 and Figure 3).

**FIGURE 4:**
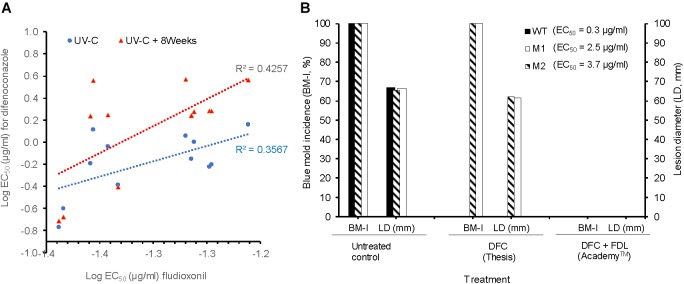
*In vitro* and *in vivo* sensitivity of *P. expansum* DIF-mutants to fludioxonil. **(A)** Correlation between the LogEC_50_ values of fludioxonil and difenoconazole for 12 isolates, i.e., the three WT-isolates and 9 resulting mutants immediately following UV-C excitation (blue) or UV-C followed by 8 weeks of selection pressure on MEA amended with DIF at 2.5 μg/ml (red). **(B)** Efficacy of DIF alone (Thesis) and DIF+FDL (Academy ™) to control the WT-isolate Pe3175 and its mutants Ml and M2 after 6 months of storage at 1°C. The efficacy was expressed as blue mold incidence (BM-I%) and lesion diameter (LD, mm). Data are the mean of 16 values across 2 experimental runs for each isolate.

The WT isolates Pe3334, Pe3136, and Pe3175 had an EC_50_ of 0.04, 0.04, and 0.05 μg/ml for fludioxonil (FDL), respectively (data not shown). The EC_50_ values of the DIF-mutants for FDL were similar to those of the WT isolates and ranged from 0.04 to 0.06 μg/ml. There was a moderate positive correlation (*R*^2^ = 0.4257) between EC_50_ values of FDL and DIF for DIF-mutants adapted for 8 weeks on DIF at 2.5 μg/ml compared to the original UV mutants (*R*^2^ = 0.3567) (Figure 4A). DIF (Thesis) alone controlled the WT isolate Pe3175 but failed to control its two mutants M1 and M2, whereas FDL (pre-mixed with DIF) in Academy™, applied preventively, fully controlled the WT isolate and its DIF-mutants after 6 months of storage at 1°C (Figure 4B).

### Sequence Analysis and Expression of the *PeCYP51* Gene

**FIGURE 5:**
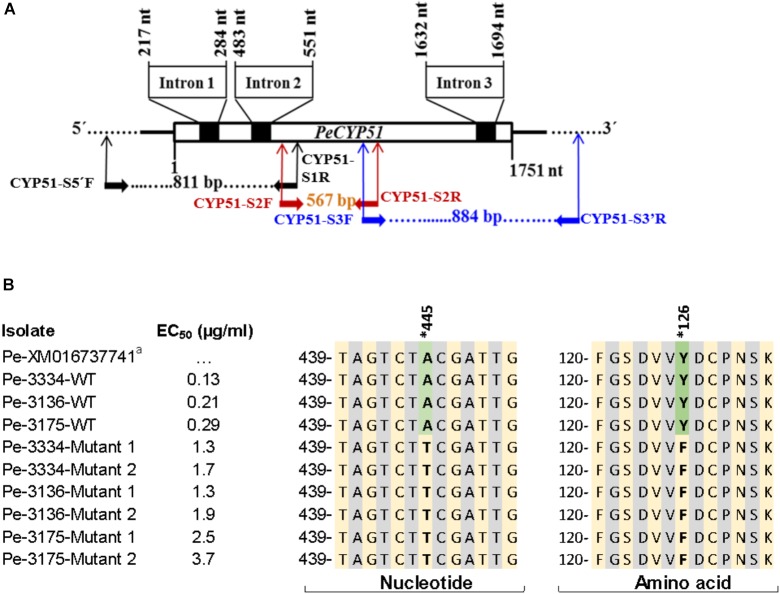
Sequencing and nucleotide and amino acid sequence alignment of *PeCYP51.* **(A)** Schematic representation of the *CYP51* gene of *Penicillium expansum* including the positions of the introns and primer binding sites used for *CYP51* sequencing. **(B)** Nucleotide and amino acid sequences alignment of *P. expansum* isolates, with different EC_50_ values, i.e., 3 wild-type and 2 resulting mutants from each WT with the reference GenBank sequence accession # XM016737741. Asterisk indicates the nucleotide and codon (or equivalent) where a mutation has been seen and reported previously to confer resistance to DMI fungicides.

The sequencing of full *PeCYP51* gene of *P. expansum* yielded a sequence with a length of 1751 bp with three introns of 68, 69, and 63 bp, respectively, and coded for 516 amino acids (Figure 5A and Supplementary Figure S2). A blast of the amino acid sequence of the WT isolate Pe3175 revealed 100, 96, 92, and 67% identity with *P. expansum, P. italicum, P. digitatum*, and *Aspergillus fumigatus* accession numbers XP106598797, KGO74727, XP014532172, and ARS45267, respectively. The alignments of nucleotide and amino-acid sequences of the GenBank reference accession number XM016737741, the sensitive WT parental isolates, and the resulting DIF-mutants is shown in Figure 5B. A single polymorphism from A to T at nucleotide 445 resulted in an amino-acid substitution from tyrosine to phenylalanine at codon 126 (Y126F) of the *PeCYP51* gene was detected in all mutants regardless of their EC_50_ value and was absent in all WT isolates (Figure 5B and Supplementary Figure S2). The *CYP51* sequence from the Pe3175 WT-isolates and of its mutants (M1) were submitted to GenBank under the accession numbers MH507024 and MH507025, respectively.

**FIGURE 6:**
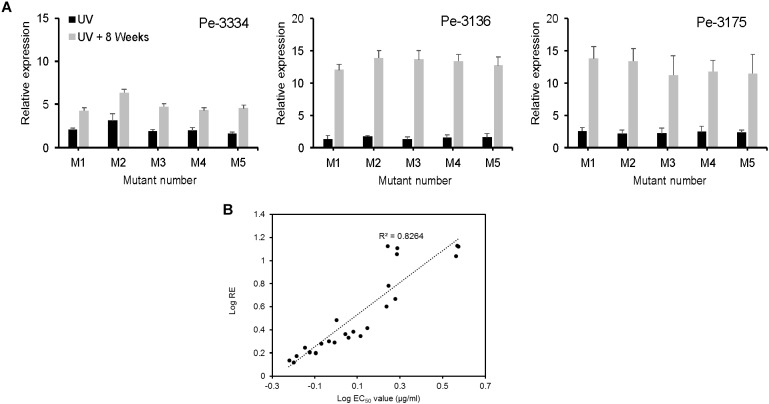
Constitutive expression of *PeCYP51* in *Penicillium expansum* and correlation with isolates sensitivity. **(A)** The relative expression was calculated with the reference β-actin gene using the 2^-ΔΔCt^ method for 5 mutants from each of the three WT-isolates Pe3334, P3136, and Pe3175 after UV excitation (black columns) or after 8 weeks of selection pressure on MEA amended with 2.5 μg/ml (gray columns). The EC_50_ values of the mutants are shown in Table 1. The final relative expression (RE) was expressed as the RE in the mutant relative to the RE in the respective wild-type isolate. **(B)** Correlation between the LogEC_50_ values of the *P. expansum* mutant isolates and their relative *CYP51* expression.

We evaluated the *CYP51* gene expression in 3 parental and 5 mutant isolates not challenged with DIF prior to RNA extraction. The relative expression (RE) of the *CYP51* gene increased 2 to 3 folds in UV-mutants and 4 to 14 fold in mutants adapted for 8 weeks DIF at 2.5 μg/ml (Figure 6A). The *CYP51*-RE was positively and significantly correlated (*R*^2^ = 0.8264) with the EC_50_ values of the isolates (Figure 6B). The mutants resulting from the Pe-3334 WT-isolate (EC_50_ = 0.21 μg/ml) had a lower *CYP51* RE compared to the mutants from the two other isolates after UV excitation or adaptation on DIF (Figure 6A).

## Discussion

Demethylation inhibitors have been used for years to control *P. digitatum* and *P. italicum* and other citrus pathogens. However, difenoconazole (DIF) is the first DMI registered for managing *P. expansum* and other postharvest diseases of pome fruit. The baseline *P. expansum* population was highly sensitive to DIF, as shown by the low EC_50_ values (<0.5 μg/ml) for all growth stages, i.e., germination, germ tube elongation, and mycelial growth, *in vitro* as well by the ability of DIF to fully control blue mold infections on apple fruits for at least 6 months in cold storage. High control efficacy of DIF has been reported in several ascomycetes such as *Phacidiopycnis* spp., *Venturia inaequalis, Colletotrichum* spp., *Marssonina coronaria, Alternaria* spp., and *Fusarium* spp. (Munkvold and O’Mara, 2002; Villani et al., 2015; Fonseka and Gudmestad, 2016; Cao et al., 2017; Dang et al., 2017; Ali et al., 2018) and the basidiomycete *Rhizoctonia solani* (Bartholomäus et al., 2017). Given the widespread occurrence of resistance to two of the three current postharvest fungicides and the stringent limitations to new postharvest fungicides registration, DIF could be a valuable additional tool to manage blue mold of pome fruit in the years to come if used appropriately and if its efficacy against other major postharvest diseases is proven.

Our data suggest a risk for *P. expansum* to develop resistance to DIF in packinghouses where it is expected to be part of regular management programs. This suggests it should be rotated with other fungicides, to extend its lifespan. The selection and characterization of *P. expansum* mutants in this study will be valuable to estimate the risk and the speed of DIF resistance development and will serve as a reference for future DIF resistance monitoring in exposed populations. Mutants with an EC_50_ value >0.8 μg/ml were not controlled by the DIF label rate on detached fruit, and we, therefore, suggest a dose of 1.0 μg/ml and above as a potential discriminatory dose for future DIF resistance monitoring. Fitness penalties have been linked with resistance to other DMIs in multiple pathogens such as *Monilinia fructicola, Aspergillus nidulans*, and *Colletotrichum truncatum* (Van Tuyl, 1977; Chen et al., 2012; Zhang et al., 2017) but not in *P. digitatum* (Nakaune et al., 2002). We did not investigate the fitness of the DIF-*P. expansum* mutants, but the latter were as virulent at the parental wild-type isolates on apple fruit in the absence of a DIF selection pressure (Table 1). Moreover, although the level of resistance to DIF in the mutants slightly decreased over a 10-week period in the absence of a selection pressure *in vitro*, the EC_50_ values remained within the resistance range. Given that *P. expansum* is considered among the group of fungi with a “high risk” for fungicide resistance development, field resistance is likely to occur and persist in packinghouses if rational practices are not implemented immediately upon registration.

The mixture of DIF with fludioxonil as Academy™ should be effective in controlling DIF-resistant populations of *P. expansum* if/when they emerge in commercial packinghouses. Indeed, no cross-resistance was observed between FDL and DIF and the EC_50_ values (≤0.06 μg/ml) of the DIF-mutants for FDL were not different from those of the parental isolates. There was a correlation with *in vivo* susceptibility as the label rate of Academy™ fully controlled the DIF-resistant mutants on apple fruit after 6 months of storage (Figure 4B). However, because of a slightly stronger positive correlation (Figure 4A) observed between the EC_50_ values of FDL and DIF under a DIF-continuous selection pressure, further investigations are needed to ensure that mechanisms of resistance in *P. expansum* do not select for dual-resistant populations as it has been reported recently in the closely related species *P. digitatum* (Wang et al., 2014).

We present evidence that resistance to DMIs in *P. expansum* is likely caused by variation in the amino acid sequence and overexpression of the *PeCYP51* gene, although other mechanisms cannot be completely excluded. The Tyr-Phe mutation found at codon 126 of *P. expansum* is well known for its role in resistance to DMIs as an equivalent mutation at codon 136 of *Erysiphe necator* (Délye et al., 1997, 1998; Frenkel et al., 2015), *Blumeria graminis* (Wyand and Brown, 2005), and *Parastagonospora nodorum* (Pereira et al., 2017) was reported to confer resistance to different DMIs. Other amino acid substitutions at different codons have also been reported to cause DMI resistance in several other plant pathogens (Leroux et al., 2007; Wang et al., 2015; Mair et al., 2016; Lichtemberg et al., 2017; Pereira et al., 2017). The Y126F substitution was present in all DIF mutants with an EC_50_ value >1.0 μg/ml and no other mutation was detected in mutants with higher EC_50_ values (>3 μg/ml) after 8 weeks of selection pressure which suggest a major role of this alteration in conferring resistance to the DMI fungicides in *P. expansum*. If a single point mutation is proven to be the major driving factor of resistance to DIF and other DMIs in *P. expansum*, resistance can be expected to emerge and build-up quickly once DIF is used frequently in the packinghouses.

The Y126F alteration is located in the conserved substrate binding domain of the *CYP51* gene (van Nestelrooy et al., 1996) and a mutation in this region could affect mRNA stability and heme structure of *CYP51* which can decrease the affinity of DMIs as reported in *Candida albicans* (Kelly et al., 1999). The reduced affinity due to smaller amounts fungicide docking to the binding site prevents complete control. The significant 12 to 14-fold increase in the expression of the *CYP51* gene without DIF induction prior to total RNA extraction in mutants of the second generation (selection pressure) suggests a role of *CYP51* overexpression in DMI resistance in *P. expansum*. In the closely related species *P. italicum* and *P. digitatum*, several mechanisms of resistance to DMIs have been elucidated. Thus, the expression of the *P. italicum-CYP51* gene by heterologous combination in *Aspergillus niger* was 2 to 5-fold higher in a resistant transformant compared to a wild-type isolate, but whether a change in the amino-acid sequence has occurred was not investigated (van Nestelrooy et al., 1996). In the citrus green mold-causal species *P. digitatum*, resistance to DMIs has been linked to the *ABC* and *CYP51* genes. *Penicillium* multidrug resistance (*PMR1* and *PMR5*) genes encoding an ATP-binding cassette were suggested to play a role in *P. digitatum* resistance to the DMIs (Nakaune et al., 1998, 2002; Sun et al., 2013) although the role of *PMR1* was not clearly evidenced by Hamamoto et al. (2001b). Moreover, a 199 bp transposon insert in the promoter region of the *CYP51* gene of *P. digitatum* DMI-resistant isolates increased its expression 7.5 to 13.6-fold (Ghosoph et al., 2007; Sun et al., 2011), similar to the overexpression levels seen in *P. expansum* mutants in this study. Recently, a role of major facilitator superfamily transporters (MFS) has been hypothesized as potential mechanisms of DMI-resistance in *P. digitatum* (Wu et al., 2016). Worrisomely, some of the above mechanisms reported in *P. digitatum* were also found to confer multidrug (MDR) resistance (Nakaune et al., 2002; Sun et al., 2011). The 100 bp sequenced upstream and downstream the *CYP51* of *P. expansum* did not reveal any mutations in the DIF-mutants (data not shown). Research investigation is ongoing to explore additional potential mutations and study a potential role of the above or other mechanisms in DMI or MDR resistance of *P. expansum* populations in commercial packinghouses. This information will be critical to clearly assess the expected risk for resistance emergence in this pome fruit-*Penicillium* pathosystem and develop appropriate management strategies.

In summary, we conducted a risk assessment study to evaluate the efficacy and risk associated with the introduction of a new DMI in the pome fruit-postharvest system. We showed a high and lasting control efficacy of difenoconazole alone or in combination with fludioxonil. However, resistance to DIF seems likely to emerge in *P. expansum* packinghouse populations for which resistance levels and speed of selection will depend on the actual mechanism(s) of resistance and the selection pressure through usage frequency.

## Author Contributions

AA designed the project and supervised the work. EA performed the experiments and analyzed the data. All authors participated in writing and editing the manuscript.

## Conflict of Interest Statement

The authors declare that the research was conducted in the absence of any commercial or financial relationships that could be construed as a potential conflict of interest.

## Footnotes

1 www.bio-rad.com
