# Supplementary material for: Selection Pressure Pathways and Mechanisms of Resistance to the Demethylation Inhibitor-Difenoconazole in Penicillium expansum
Source: Front Microbiol. 2018 Oct 31;9:2472. doi: 10.3389/fmicb.2018.02472 (PMC6220093; doi:10.3389/fmicb.2018.02472)
Supplement: Supplementary file 1 [file Table_1.docx]

**SUPPLEMENTARY MATERIAL**

**TABLE 1S** Primers used in this study to characterize the *P. expansum* isolates, sequence and measure the relative expression of the *PeCYP51* gene

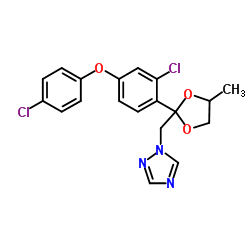

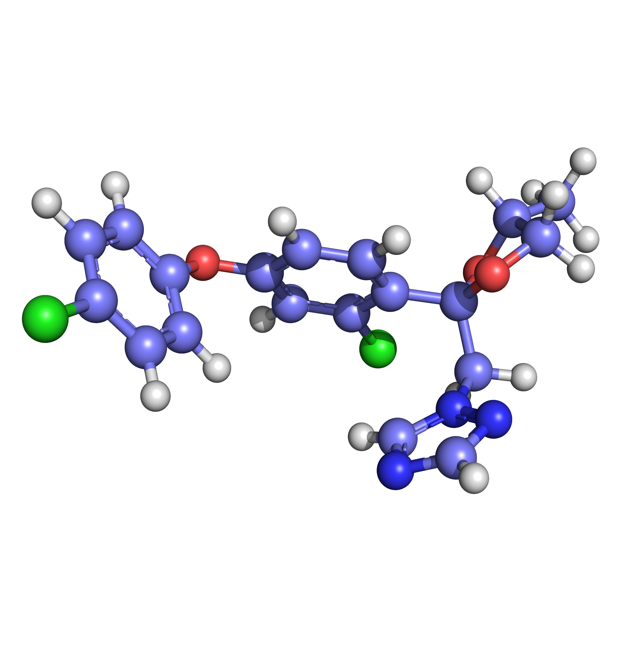


**FIGURE S1** Chemical structure of difenoconazole

**
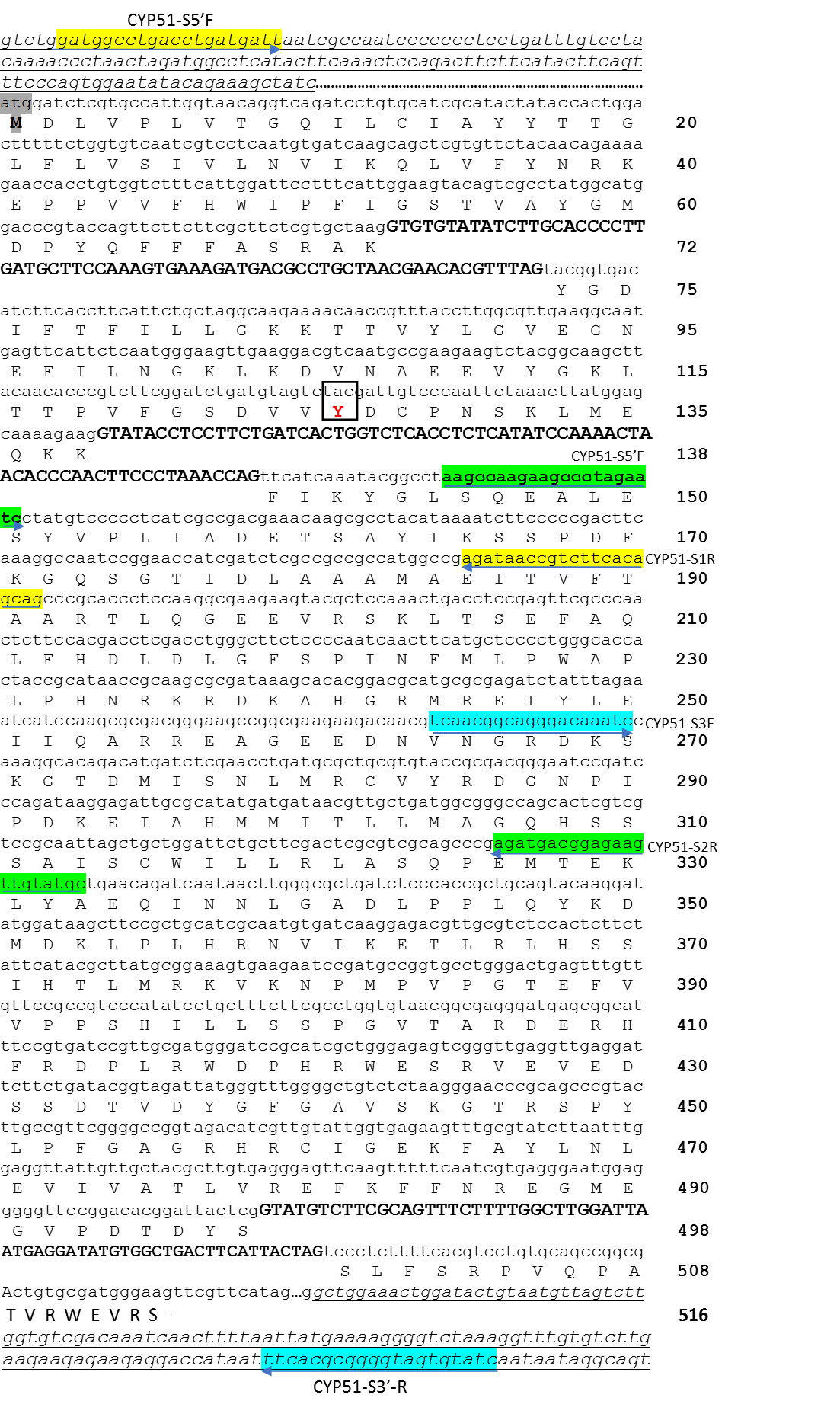
**

**FIGURE S2** Nucleotide and deduced amino acid (single capital letter below the nucleotides) sequence of *Penicillium expansum* *PeCYP51* gene of the Pe3175 wild-type isolate. Start codon of the *CYP51* is shaded in gray and codon numbering was based on the GenBank reference sequences with accession numbers XM016737741 and NW015971309. Exons are in lower case and introns are in bold upper case. The position of the primers used for *CYP51* sequencing are shown in shaded color of same color for each primer set. The box indicates codon at which a mutation of tyrosine (Y) has been reported to confer resistance to the DMIs. The underlined nucleotides upstream (143 nucleotides) and downstream (142 nucleotides) the *CYP51* gene were added from the GenBank sequence NW015971309 and used to design primers to sequence the full *PeCYP51* gene.
